# Glycosylation at Asn254 Is Required for the Activation of the PDGF-C Protein

**DOI:** 10.3389/fmolb.2021.665552

**Published:** 2021-05-24

**Authors:** Wenjie Hu, Ruting Zhang, Wei Chen, Dongyue Lin, Kun Wei, Jiahui Li, Bo Zhang, Xuri Li, Zhongshu Tang

**Affiliations:** ^1^ State Key Laboratory of Ophthalmology, Zhongshan Ophthalmic Center, Sun Yat-Sen University, Guangzhou, China; ^2^ The First Affiliated Hospital of Guangzhou University of Traditional Chinese Medicine, Guangzhou University of Chinese Medicine, Guangzhou, China

**Keywords:** platelet-derived growth factor, glycosylation, PDGF receptors, HEK 393A cells, NIH 3T3 cells, site-directed mutagenesis, ER, golgi apparatus

## Abstract

Platelet-derived growth factor C (PDGF-C) is a member of the PDGF/VEGF (vascular endothelial growth factor) family, which includes proteins that are well known for their mitogenic effects on multiple cell types. Glycosylation is one of the most important forms of posttranslational modification that has a significant impact on secreted and membrane proteins. Glycosylation has many well-characterized roles in facilitating protein processing and contributes to appropriate folding, conformation, distribution, and stability of proteins that are synthesized intracellularly in the endoplasmic reticulum (ER) and Golgi apparatus. Although the general process and functions of glycosylation are well documented, there are most likely others yet to be discovered, as the glycosylation of many potential substrates has not been characterized. In this study, we report that the PDGF-C protein is glycosylated at three sites, including Asn25, Asn55, and Asn254. However, we found that mutations at any of these sites do not affect the protein expression or secretion. Similarly, disruption of PDGF-C glycosylation had no impact on its progression through the ER and Golgi apparatus. However, the introduction of a mutation at Asn254 (N254 A) prevents the activation of full-length PDGF-C and its capacity for signaling *via* the PDGF receptor. Our findings reveal that glycosylation affects PDGF-C activation rather than the protein synthesis or processing. This study characterizes a crucial modification of the PDGF-C protein, and may shed new light on the process and function of glycosylation.

## Introduction

Platelet-derived growth factor C (PDGF-C) is a member of the PDGF/VEGF (vascular endothelial growth factor) family. Proteins in this family include eight Cys residues and a cystine knot (Li et al., 2000). Unlike other members of this family, full-length PDGF-C (PC-FL) protein is composed of two domains, including an N-terminal complement C1r/C1s, Uegf, Bmp1 (CUB) domain, and a C-terminal core domain (PC core) (Fredriksson et al., 2004). PDGF-D is the only other member of this family that has a CUB domain (LaRochelle et al., 2001). Most other family members have the core domain only.

Previous work has established that PC-FL undergoes extracellular activation and cleavage that results in the release of the core domain. The PC core can then interact with PDGF receptors (PDGFRs) α and β (Gilbertson et al., 2001; Fredriksson et al., 2005; Hurst et al., 2012; Lee and Li, 2018). PC-FL activation is regulated by extracellular proteases, including tissue plasminogen activator. PDGF-C has been shown to play critical roles in embryonic development, fibrosis, wound healing, and angiogenesis (Ding et al., 2000; Cao et al., 2002; Grazul-Bilska et al., 2003; Li et al., 2005; Crawford et al., 2009). It is also considered to be a potential target for the development of antifibrotic strategies as well as therapies that might be used to treat ocular diseases and cancer (Ponten et al., 2003; Tang et al., 2010; Manzat Saplacan et al., 2017; Klinkhammer et al., 2018; Kumar and Li, 2018; Bottrell et al., 2019).

Glycosylation is a posttranslational modification in which sugar chains are covalently linked to a substrate *via* asparagine (i.e., N-linked glycosylation) or serine/threonine/tyrosine (i.e., O-linked glycosylation) residues (Medzihradszky, 2008; Lee et al., 2015; You et al., 2018; Eichler, 2019). This modification contributes to a variety of biological functions that include but are not limited to cell-cell interactions, signal transduction, and immunogenicity (Mitra et al., 2006; Pearse and Hebert, 2010; Lu et al., 2012; Dewald et al., 2016). Nearly all membrane and secreted proteins are likely to be glycosylated, with rare exceptions (Stowell et al., 2015). Alterations in protein glycosylation may disrupt glycoprotein functions (Stowell et al., 2015). Thus, it is not surprising that dysfunctional glycosylation may promote various diseases, including cancer, infection, and neurodegeneration (Freeze et al., 2015; Stowell et al., 2015; Nagel et al., 2018; Moll et al., 2020).

While three potential glycosylation sites were originally proposed in PC-FL when it was discovered (Li et al., 2000), none of these sites have been verified or characterized. In this study, we aimed to examine the glycosylation of PDGF-C. Among these experiments, we performed deglycosylation studies of PDGF-C in mouse tissue samples, located the glycosylation sites, and explored the impact of glycosylation on PDGF-C–mediated activities.

## Materials and Methods

### Molecular Cloning

Recombinant human (rh)*PDGF-C* (Vigenebio, China) was inserted into a site upstream of the *IRES* in the plasmid vector pAAV-IRES-ZsGreen (Biowit Technologies Ltd., China). Site-directed mutagenesis was performed to construct Asn→Ala (NA) mutations at potential PDGF-C glycosylation sites. All constructs were verified by DNA sequencing. The expression and sizes of the recombinant proteins were verified by Western blot (see Results).

The sequences of the site-directed mutagenesis primers are as follows:

PDGF-C-N25A-s: 5′ GGG​ACT​CAG​GCG​GAA​AGC​GCT​CTG​AGT​AGT​AAA​TTC 3′
PDGF-C-N25A-as: 5′ GAA​TTT​ACT​ACT​CAG​AGC​GCT​TTC​CGC​CTG​AGT​CCC 3′
PDGF-C-N55A-s: 5′ ATT​ATT​ACT​GTG​TCT​ACA​GCT​GGA​AGT​ATT​CAC​AGC 3′
PDGF-C-N55A-as: 5′ GCT​GTG​AAT​ACT​TCC​AGC​TGT​AGA​CAC​AGT​AAT​AAT 3′
PDGF-C-N254A-s: 5′ TAC​AGC​TGC​ACA​CCT​AGG​GCC​TTC​TCA​GTG​TCC​ATA 3′
PDGF-C-N254A-as: 5′ TAT​GGA​CAC​TGA​GAA​GGC​CCT​AGG​TGT​GCA​GCT​GTA 3′
PDGF-C-N290A-s: 5′ GCC​TGT​TGT​CTC​CAC​GCA​TGC​AAT​GAA​TGT​CAA​TG 3′
PDGF-C-N290A-as: 5′ CAT​TGA​CAT​TCA​TTG​CAT​GCG​TGG​AGA​CAA​CAG​GC 3′

### Cell Culture

HEK293 A cells and NIH 3T3 cells from the American Type Tissue Collection (ATCC, Manassas, VA, United States) were cultured in Dulbecco’s Modified Eagle Medium (DMEM; ExCellBio, China) supplemented with 10% fetal bovine serum (FBS; ExCellBio, China) and 1% pen/strep (Cellgro, United States) at 37°C in a 5% CO_2_ incubator. Cells were passaged one day before they were subjected to various treatments.

### Transfection

Plasmids were transfected into HEK293 A cells with Lipofectamine 2000 (Invitrogen) according to the manufacturer’s protocols. In brief, plasmids were mixed with Lipofectamine 2000 in Opti-MEM medium and incubated for 5 min at room temperature to allow for complex formation. The complex was then added to cell cultures in a complete culture medium as described above. Cells were incubated for 48 h before further evaluation and/or use in experimental protocols.

### Western Blot

Fresh tissues or cultured cells were lysed in radioimmunoprecipitation assay (RIPA) buffer containing protease and phosphatase inhibitor cocktails (ThermoFisher Scientific). Samples were quantitated with a DC™ Protein Assay Kit (Bio-Rad), separated on an 8–12% sodium dodecyl sulfate (SDS)–polyacrylamide gel electrophoresis (PAGE) matrix, and transferred to a polyvinylidene difluoride (PVDF) membrane (Bio-Rad). After blocking with 5% bovine serum albumin (BSA), the membrane was incubated with a primary antibody at 4°C overnight, followed by incubation with horseradish peroxidase (HRP)–conjugated secondary antibody (1:5,000) for 1 h at room temperature. Each step was followed by three washes with Tris-buffered saline buffer with Tween-20 (TBST), after incubation. Primary antibodies included goat anti-PDGF-C (core domain; 1:1,000, R&D Systems, AF1447), rabbit anti-ZsGreen (1:1,000, Takara, 632,474), rabbit anti-*p*-PDGFRα Tyr762 (1:1,000, Cell Signaling Technology (CST), 24,188), rabbit anti-PDGFRα (1:750, Abcepta, AP7666d), rabbit anti-PDGFRβ (1:1,000, CST, 3,169), rabbit anti-*p*-PDGFRβ Tyr1009 (1:1,000, CST, 3124s), mouse-anti-glyceraldehyde 3-phosphate dehydrogenase (GAPDH; Beijing Ray Antibody Biotech), and mouse-anti-β-actin (Beijing Ray Antibody Biotech). Immobilon Western Chemiluminescent HRP substrate (Merck Millipore) was used to detect specific antibody binding. These signals were captured using a G-BOX (Syngene) imaging system or a LI-COR Odyssey Sa system (LI-COR Biosciences).

### Deglycosylation Assay

A Native Protein Deglycosylation Kit (Sigma, NDEGLY-1KT) and PNGase F (New England Biolabs) were used to detect protein glycosylation. The Native Protein Deglycosylation Kit was also used to identify the various types of glycosylation. This kit contains three enzymes, including endoglycosidase F3 (removes triantennary and trimannosyl chitobiose core structures), endoglycosidase F2 (removed biantennary structures), and endoglycosidase F1 (targets oligomannose and hybrid structures). The enzymatic assays were conducted according to the product manuals.

### PDGF-C Secretion

HEK293 A cells were transfected using Lipofectamine 2000 (Invitrogen). Two days later, the cells were washed in phosphate buffered saline (PBS) and were maintained in serum-free medium. Conditioned medium (CM) was collected after 6 h in culture. Debris was removed by centrifugation. CMs were concentrated by Amicon Ultra filtration device (Merck Millipore UFC801096) before analysis for PDGF-C expression by Western blot.

### PDGF Receptor Activation

NIH 3T3 cells were maintained in a serum-free medium overnight before the addition of CM collected from transfected HEK293 A cells as described above. After 5 min of incubation with CM, NIH 3T3 cells were collected, lysed in RIPA buffer containing protease and phosphatase inhibitor cocktails (ThermoFisher Scientific), and analyzed with anti-*p*-PDGFRα Tyr762 and anti-*p*-PDGFRβ Tyr1009 by Western blot as described above.

### Immunofluorescence Staining

Immunofluorescence was performed on cryostat-generated sections of spleens from C57BL/6 mice and transfected cells that were plated on glass coverslips. Tissues and cells were washed briefly with PBS and fixed for 15 min in 4% paraformaldehyde. After three washes with PBS, samples were pretreated for 1 h in PBS supplemented with 5% donkey serum and 0.5% Triton X-100. Cells and tissues were then incubated overnight at 4°C with the primary antibody in PBS supplemented with 5% donkey serum and 0.1% Triton X-100 and Alexa Fluor–conjugated secondary antibodies (1:500, Invitrogen) for 1 h. Then, 0.1% DAPI (4′,6-diamidino-2-phenylindole) was applied for 5 min followed by three washes. Primary antibodies included goat anti–PDGF-C core domain (1:100, Abcam, AF1447), mouse anti-ERP72 (1:50, Proteintech, 66,365–1), and sheep anti-TGN46 (1:100, Bio-Rad, AHP500). Cells were observed under a confocal microscope (LSM710, Carl Zeiss).

## Results

### PDGF-C Protein Is N-Glycosylated at Three Sites

**FIGURE 1:**
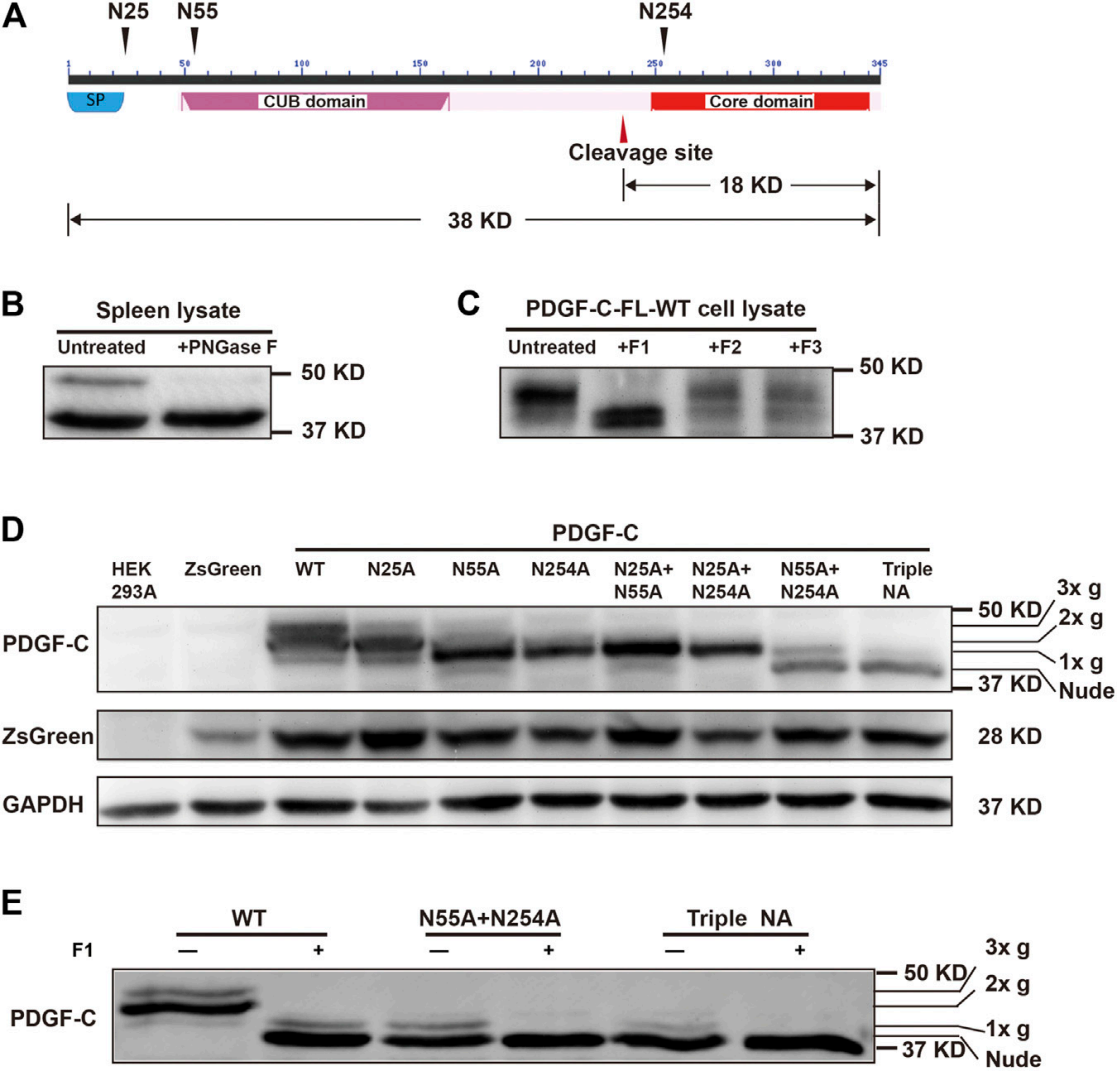
PDGF-C is N-glycosylated at Asn25, Asn55, and Asn254. **(A)** Structure of the PDGF-C protein. PDGF-C is composed of two domains, including the CUB domain and the core domain. Three putative glycosylation sites and a putative cleavage site are as indicated. The molecular weights of full-length PDGF-C and the PDGF-C core domain are 38 and 18 kDa, respectively. **(B)** Deglycosylation of endogenous PDGF-C. Mouse spleen lysates were treated with PNGase F and evaluated by Western blotting with a primary antibody directed against the PDGF-C core domain. The control sample was treated identically without the enzyme. **(C)** Deglycosylation of the full-length PDGF-C. Plasmids encoding full-length PDGF-C were transfected into HEK293 A cells. Cell lysates were analyzed with a Native Protein Deglycosylation Kit to characterize the type of glycosylation. **(D)** Expression of the full-length PDGF-C mutant proteins in HEK293 A cells. Cell lysates were analyzed with an antibody that detects the PDGF-C core domain; antibodies that detected ZsGreen and GAPDH were used as controls. 3x g, 2x g, and 1x g represent triple, double, and single glycan chains, respectively. **(E)** Deglycosylation of full-length PDGF-C mutants detected in transfected HEK293 A cell lysates.

We identified three potential N-linked glycosylation sites that were conserved in human, rat, and mouse PC-FL that included Asn25, Asn55, and Asn254 (https://services.healthtech.dtu.dk/service.php?NetNGlyc-1.0). No O-linked glycosylation sites were predicted (https://services.healthtech.dtu.dk/service.php?NetOGlyc-4.0). Both Asn25 and Asn55 were located in the CUB domain of PC-FL. Of these, Asn25 immediately followed a predicted 22-amino acid signal peptide. Asn254 was located in the core domain and was situated near the cleavage site for the activation of PDGF-C protein (Figure 1).

To confirm these predictions, we performed a deglycosylation assay. Lysate of mouse spleen tissue were treated with PNGase F and analyzed by Western blotting. The bands were detected with an anti-PDGF-C antibody. We identified two immunoreactive bands in the untreated lysate. The band migrating at ∼50 kDa molecular weight disappeared after PNGase F treatment. This result suggests that PDGF-C is a glycoprotein (Figure 1B).

Next, we subcloned the *PC-FL* sequence upstream of the IRES in the pAAV-IRES-ZsGreen plasmid to express rhPDGF-C in mammalian cells. We selected HEK293 A cells for the heterologous expression of PDGF-C because we were unable to detect any endogenous PDGF-C expression in these cells in our pilot test. We used a native protein deglycosylation kit to characterize the nature of N-linked glycosylation. We found that the high molecular weight band disappeared in response to the treatment with endoglycosidase F1 but not with endoglycosidases F2 or F3 (Figure 1C). These results suggest that PDGF-C is substituted with high mannose-containing glycoprotein.

We then generated NA mutants to locate the specific glycosylation sites. For this experiment, the three predicted Asn residues were mutated to Ala and evaluated in single, double, or triple combinations. Wild-type and mutant constructs were transfected into HEK293 A cells to evaluate the size of the PDGF-C proteins and their glycosylation state.

As shown in Figure 1D, PDGF-C proteins were detected in lysates from all mutant-transfected cells by Western blotting. Four distinct bands were detected. The wild-type (WT) protein ran as three bands that corresponded to PC-FL with three (3x g), two (2x g), or one (1x g) glycan chains. The apparent molecular weights of the main bands in all three single mutants decreased compared with that of the WT. These results suggest that all three predicted Asn residues are functional glycosylation sites.

Of the single mutants, PDGF-C-N25 A contained two glycans (2x g), whereas the N55 A and N254 A mutants each contained one glycan (1x g) only. As shown, the N55 A and N254 A single mutants displayed the same Western blot pattern as did their corresponding double mutants that also included N25 A (i.e., N25 A + N55 A, and N25 A + N254A, respectively). These results suggest that glycosylation at Asn25 requires concomitant glycosylation at Asn55 or Asn254. Alternatively, glycosylation at Asn55 and Asn254 takes place earlier than that occurring at Asn25.

The main band in the triple NA mutant is considered to be a fully unsubstituted or nude protein that contains no glycan residues. We note detection of a low intensity band suggestive of single glycan-substitution which was detected on a Western blot of the triple NA mutant. This result suggests that PDGF-C may have an additional yet an unidentified glycosylation site. To explore the possibility of one or more additional glycosylation sites, we analyzed lysates from cells transfected with the N55 A + N254 A double mutant and the triple NA mutant with the Native Protein Deglycosylation kit. As shown in Figure 1E, the low intensity bands disappeared after treatment. This result suggests that there may be additional glycosylation sites in PDGF-C.

### Glycosylation Is Not Required for the Expression and Secretion of PDGF-C Protein

**FIGURE 2:**
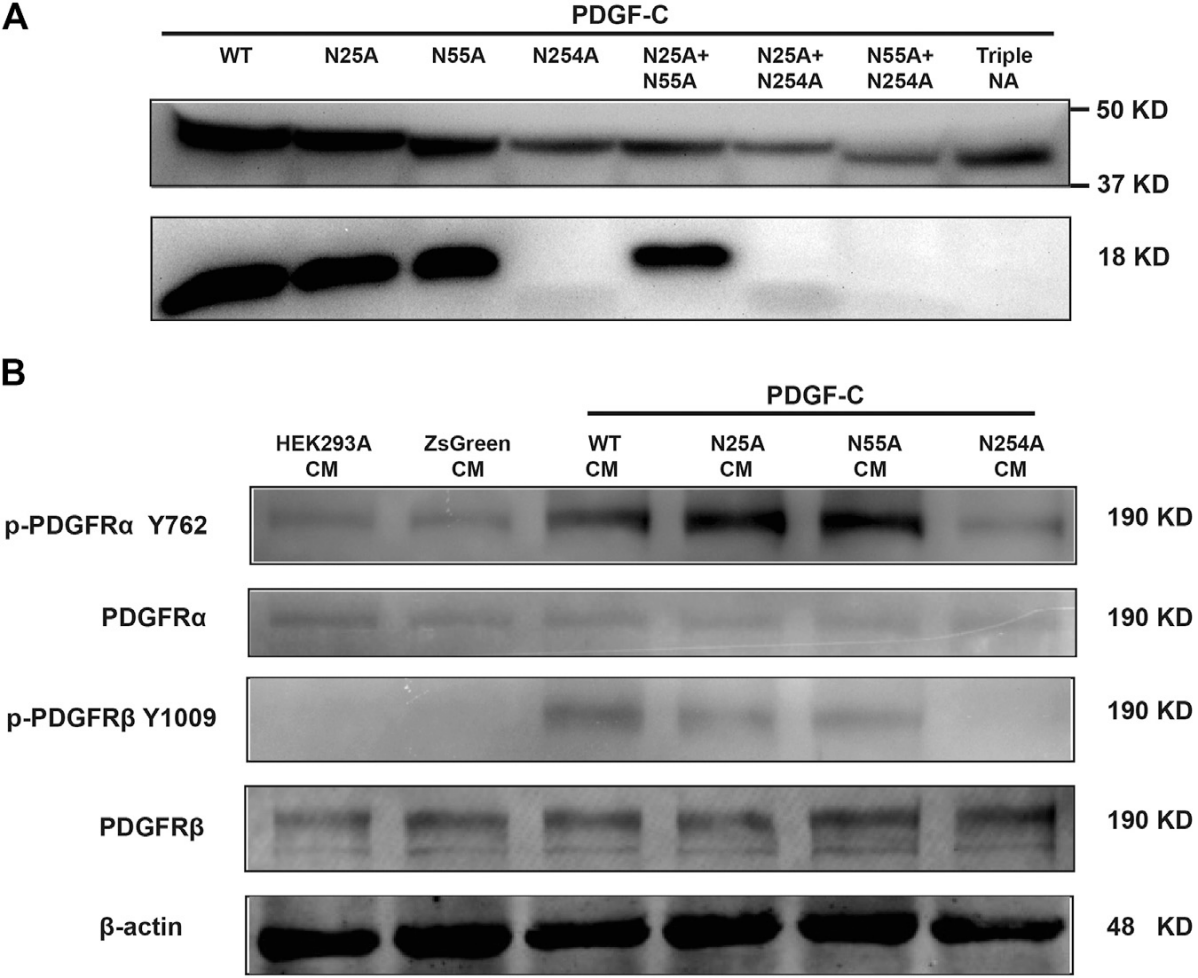
Glycosylation at Asn254 is required for the PDGF-C activation and signaling of PDGF-C. **(A)** Glycosylation at Asn254 is required for the PDGF-C activation. HEK293 A cells were transfected with plasmids carrying wild-type (WT) or mutant forms of PDGF-C. Two days later, CMs were collected, concentrated, and analyzed by Western blot with antibodies that detected full-length and the core-domain of PDGF-C. **(B)** Glycosylation at Asn254 was required for PDGF-C–mediated signaling *via* PDGFR-α and-β. CMs were collected from transfected HEK293 A cells as described in A and added to cultured NIH 3T3 cells for 5 min. NIH 3T3 cells were harvested and analyzed by Western blot.

The results discussed above indicate that both WT and NA mutants of PDGF-C proteins were detected in transfected cell lysates. In its role as a growth factor, PDGF-C is secreted and functions extracellularly to activate PDGFRs. To examine the secretion of immunoreactive PDGF-C, we collected CMs from transfected cells and analyzed protein expression by Western blot. Full-length PDGF-C proteins were detected in CMs from all cultures. Of note, PDGF-C secretion was not prevented in response to mutations introduced at any of the aforementioned glycosylation sites in single, double, or triple form (top panel in Figure 2A). This result indicates that glycosylation is not required for the expression and secretion of PDGF-C protein.

### Glycosylation at Asn254 Is Required for the Activation of the PDGF-C Protein

The results in Figure 2A were somewhat surprising because glycosylation is generally considered to be a critical modulator of protein processing. To examine the functional impact of PDGF-C glycosylation, we examined the activity of the secreted form of the protein. Once secreted, full-length PDGF-C is activated *via* proteolytic removal of the N-terminal CUB domain. The remaining PDGF-C core domain functions as a PDGFR agonist. We evaluated the core domain in CMs from WT and mutant-transfected cells. As shown in the bottom panel of Figure 2A, the core domain was detected in the CMs from both WT and N25 A and N55 A PDGF-C mutants. These results suggested that glycosylation at N25 or N55 is not required for the PDGF-C activation. By contrast, we were unable to detect the core domain in CMs from cells transfected with single, double, or triple mutants that included N254 A. These results suggest that glycosylation at N254 may be required for the activation and generation of full-length PDGF-C protein.

### Glycosylation at Asn254 Is Required for PDGF Receptor Signaling

The results presented in Figure 2A suggest that the N254 A mutant form of PDGF-C may be nonfunctional because of the absence of the core domain. To explore the functionality of PDGF-C in the absence of the core domain, we examined receptor activation, specifically in an assay designed to detect phosphorylation of PDGFR-α and PDGFR-β at Tyr762 and Tyr1009, respectively. NIH 3T3 cells were selected for this assay because of their abundant expression of PDGFRs. To perform this experiment, HEK293 A cells were transfected with plasmids carrying WT or mutant forms of PDGF-C. CMs were collected and added to cultured NIH 3T3 cells. After 5 min of exposure to the CMs, NIH 3T3 cells were collected, lysed, and analyzed by Western blot. We found that both PDGFR-α and PDGFR-β were phosphorylated in response to CMs from all HEK293 A cultures transfected with plasmids that maintained an intact Asn254 residue. No receptor activation or phosphorylation was detected in response to CMs from any of the N254 A mutant transfections (Figure 2B). Taken together, these results suggest that glycosylation at Asn254 is required for PDGF-C mediated receptor signaling.

### Disruption of Glycosylation has No Impact on the Intracellular Distribution of PDGF-C

**FIGURE 3:**
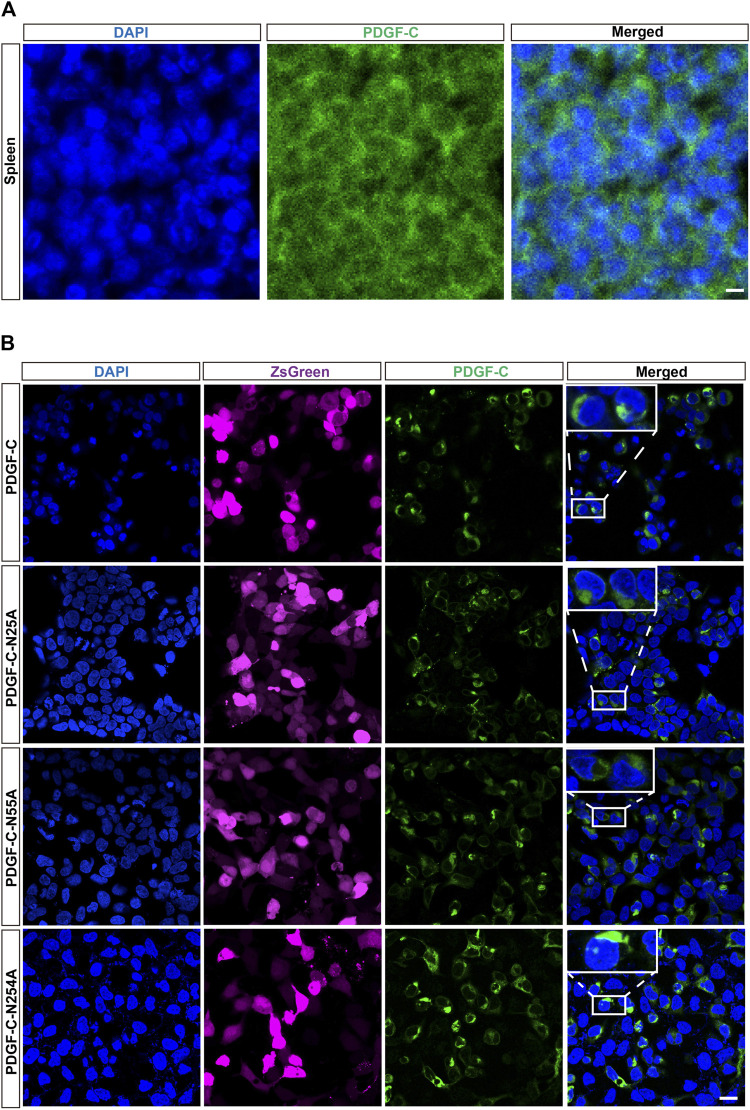
Disruption of glycosylation has no impact on the intracellular distribution of PDGF-C. **(A)** Cytoplasmic distribution of endogenous PDGF-C. Sections of mouse spleen were stained with an anti-PDGF-C core antibody. **(B)** Cytoplasmic distribution of PDGF-C protein in transfected cells. Plasmids encoding WT or NA mutant forms of PDGF-C were transfected into HEK293 A cells. The PDGF-C core antibody was used to visualize PDGF-C distribution; the ZsGreen control is colored magenta. Bar = 20 μm.

The studies discussed above focused on the detection of PDGF-C protein in cell lysates. We then assessed the impact of aberrant glycosylation patterns on the intracellular distribution of PDGF-C. The physiologic distribution of PDGF-C expression was assessed in mouse spleen tissue; immunostaining revealed that the endogenous PDGF-C was located in the cell cytoplasm (Figure 3A). Next, we evaluated the intracellular localization of PDGF-C in transfected HEK293 A cells. Untransfected HEK293 A cells expressed minimal levels of PDGF-C. Thus, most PDGF-C detected in transfected cells results from heterologous PDGF-C expression. Similar to our findings in spleen cells, immunoreactive PDGF-C (both WT and NA mutants) in transfected HEK293 A cells was also localized in the cell cytoplasm (Figure 3B). Thus, disruption of PDGF-C glycosylation sites has no apparent impact on its intracellular distribution.

### Disruption of PDGF-C Glycosylation Sites has No Impact on Its Intracellular Progression Through the ER and Golgi Apparatus

**FIGURE 4:**
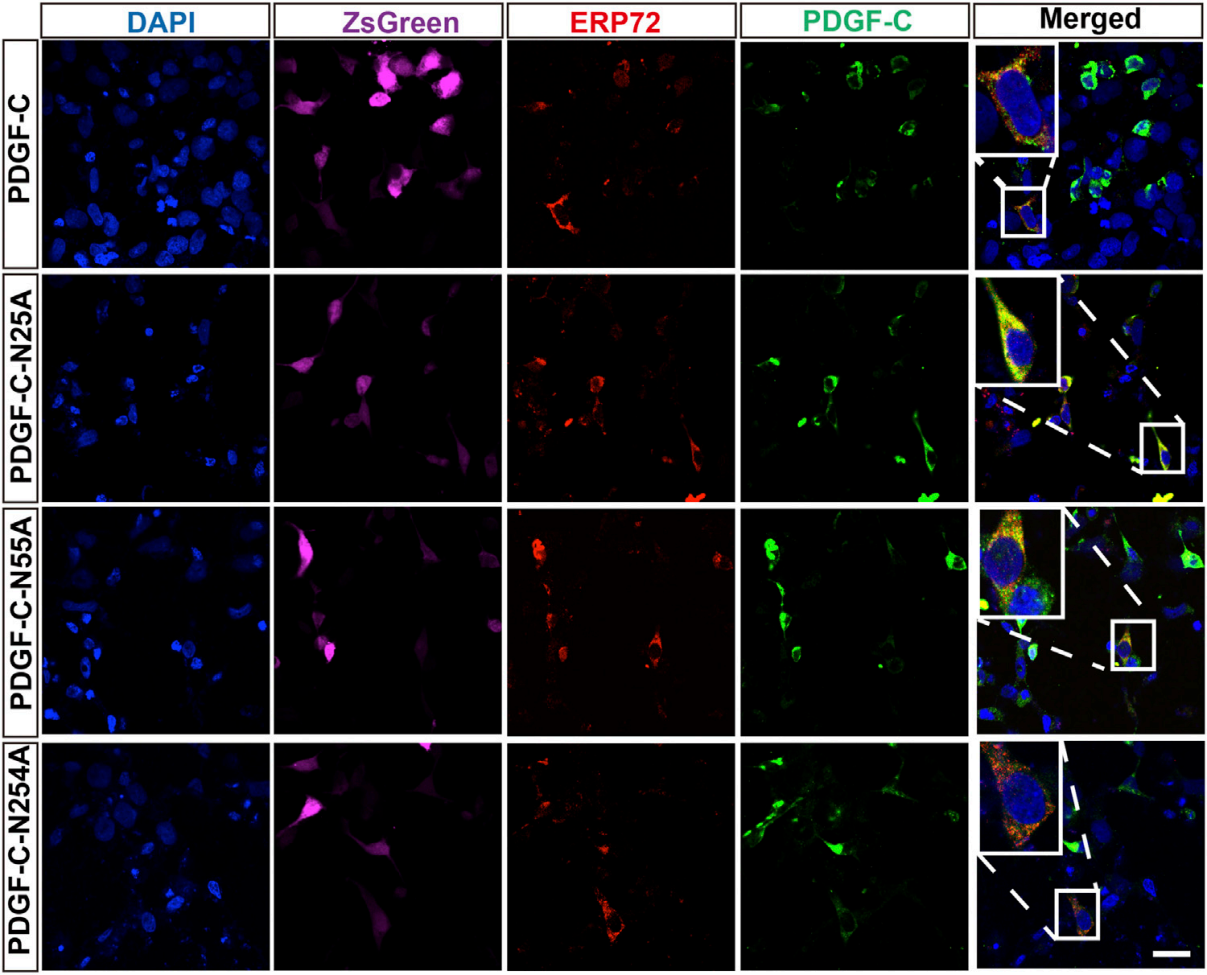
Colocalization of PDGF-C NA mutants with an ER marker. HEK293 A cells were transfected with plasmids carrying WT or NA mutant forms of PDGF-C. Anti–PDGF-C core and anti-ERP72 antibodies were used to localize PDGF-C within the ER. The ZsGreen control is colored magenta. Bar = 20 μm.

To examine the impact of glycosylation on PDGF-C protein processing, we performed immunofluorescence staining that targeted the ER and Golgi apparatus in transfected HEK293 A cells. We found that the WT PDGF-C protein was partially retained in the ER as were all of the PDGF-C proteins with single NA mutations (Figure 4). This result indicates that disruption of PDGF-C glycosylation sites does not affect its progression through the ER.

**FIGURE 5:**
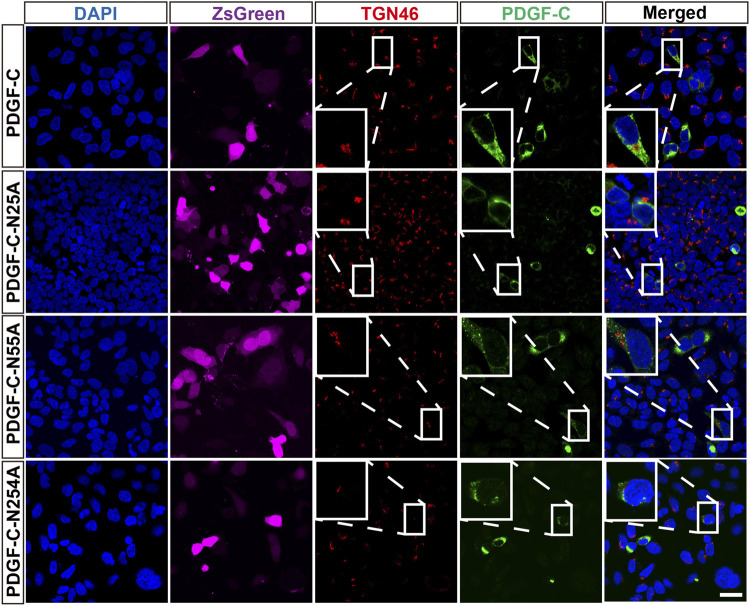
Colocalization of PDGF-C NA mutants with a Golgi marker. HEK293 A cells were transfected with plasmids carrying WT or NA mutant forms of PDGF-C. Anti–PDGF-C core and anti-TGN46 antibodies were used to localize PDGF-C in the Golgi. The ZsGreen control is colored magenta. Bar = 20 μm.

The staining pattern with respect to the Golgi apparatus was similar in both WT-transfected and untransfected cells. This pattern remained in cells transfected with each of the single NA mutants (Figure 5). These findings combined with results from the aforementioned Western blots suggest that glycosylation is not required for PDGF-C secretion; we deduce that disruption of the PDGF-C glycosylation sites has no impact on its progression through the Golgi apparatus.

## Discussion

Glycosylation is believed to make critical contributions to the protein maturation process and to provide instructions for appropriate protein folding. Protein folding begins in the ER. Further processing takes place in the Golgi apparatus *via* which mature proteins are transferred from the intracellular space to the cell surface (Stowell et al., 2015; Xu and Ng, 2015). Generally, misfolded proteins are retained in the ER and processed for ER-associated degradation (Araki and Nagata, 2012). Glycosylation deficiencies can disrupt protein localization in the ER (Zhang et al., 2019). In this study, we identified three N-glycosylation sites in the full-length PDGF-C protein that include Asn25, Asn55, and Asn254. In contrast to the generally held view that glycosylation is crucial for protein processing, we detected no disruptions in protein expression or secretion while evaluating the processing of PDGF-C with single mutations or multiple mutations at these sites. Furthermore, disruption of PDGF-C glycosylation sites does not affect its progression through the ER or the Golgi apparatus. Thus, the function of glycosylation on the PDGF-C protein might be considered to be somewhat unusual.

By contrast, our study revealed that glycosylation at Asn254 is crucial for the activation of full-length PDGF-C. We found that the N254 A mutant form of full-length PDGF-C cannot undergo proteolytic activation to generate a functional core domain. As a result, the N254 A mutant will be unable to activate PDGFR-α and PDGFR-β receptors. The Asn254 residue is located within the core domain that is conserved among all PDGF/VEGF family members. Among other PDGF/VEGF family members, we note that glycosylation patterns associated with PDGF-B have been elucidated (Kaetzel et al., 1996; Dai et al., 2015). The PDGF-B protein is comprised of a single domain that aligns with the core domain of PDGF-C and thus it does not need to undergo the activation similar to that required by PDGF-C. However, PDGF-B does undergo proteolytic processing to convert pro-PDGF-B to the mature form. Disruption of N-glycosylation on PDGF-B does not prevent its expression or secretion but it does alter the proteolytic processing (Kaetzel et al., 1996). In this respect, the N-glycosylation of PDGF-C at Asn254 and N-glycosylation of PDGF-B share similar significance. This result suggests that glycosylation may play a common role among members of this protein family.

The role played by glycosylation at N25 and N55 residues is not yet known. Both sites are located within the CUB domain. Unlike the core domain, the function of the CUB domain has not been clarified. Our understanding of the significance of glycosylation at N25 and N55 may emerge when the function of the CUB domain becomes clear.

N-Glycosylation takes place at Asn-X-Ser/Thr sites where X can be any amino acid except Pro. In rare cases, glycosylation can take place at an Asn-Pro-Cys motif (Dell et al., 2010; Xu and Ng, 2015). Except for VEGF-B, all of the other PDGF/VEGF family members contain the N-glycosylation motifs and thus may be glycosylated. Nonetheless, only a few of these glycosylation sites have been verified and characterized.

We detected an additional high molecular weight, low intensity band on the Western blot of the lysate from the cells transfected with the triple mutant. This result suggests the possibility of another glycosylation site. However, we were unable to identify additional glycosylation motifs in the amino acid sequence of PDGF-C. An additional residue might become glycosylated when the three main sites are disrupted. Human PDGF-C contains 15 Asn residues; 13 of these residues are conserved among human, rat, and mouse orthologs. Of the 13 conserved residues, PDGF-C-N290 aligns with PDGF-A-N134, which is located within an N-glycosylation motif and is conserved in all PDGF/VEGF family members. Although it is not clear whether PDGF-A-N134 is a glycosylation site, we considered the possibility that PDGF-C-N290 might be an additional glycosylation site. To explore this possibility, we generated a PDGF-C-N290 A single mutant and an N25A, N55A, N254A, and N290 A quadruple mutant. The glycosylation pattern of the PDGF-C protein was not altered upon the introduction of the N290 A mutation (Supplementary Figure S1A). This result suggests that N290 is not glycosylated. The rest nine conserved Asn residues have not yet been examined. Theoretically, any of them might serve as a site for protein glycosylation. If any one of these Asn residues is lost or mutated, any one of those remaining might serve as another substitute site. Thus, we did not examine the impact of mutations in any of the remaining nine Asn residues. We suspected that this residue was unlikely to be a part of a major glycosylation site because the high molecular weight band in the triple mutant was of very low intensity.

As PDGF-C plays a wide variety of roles in development, angiogenesis, and vascular activity, it would be intriguing to study the clinical relevance of PDGF-C glycosylation. Toward this end, we identified two PDGF-C single nucleotide polymorphisms (SNPs) with alterations within the Asn25 and Asn55 glycosylation motifs (rs747210671 and rs771283512). However, no phenotype associated with these SNPs has been reported to date.

## Data Availability

The original contributions presented in the study are included in the article/Supplementary Material, further inquiries can be directed to the corresponding author.
