# Supplementary figures and images for: Glycosylation at Asn254 Is Required for the Activation of the PDGF-C Protein
Source: Front Mol Biosci. 2021 May 24;8:665552. doi: 10.3389/fmolb.2021.665552 (PMC8181125; doi:10.3389/fmolb.2021.665552)

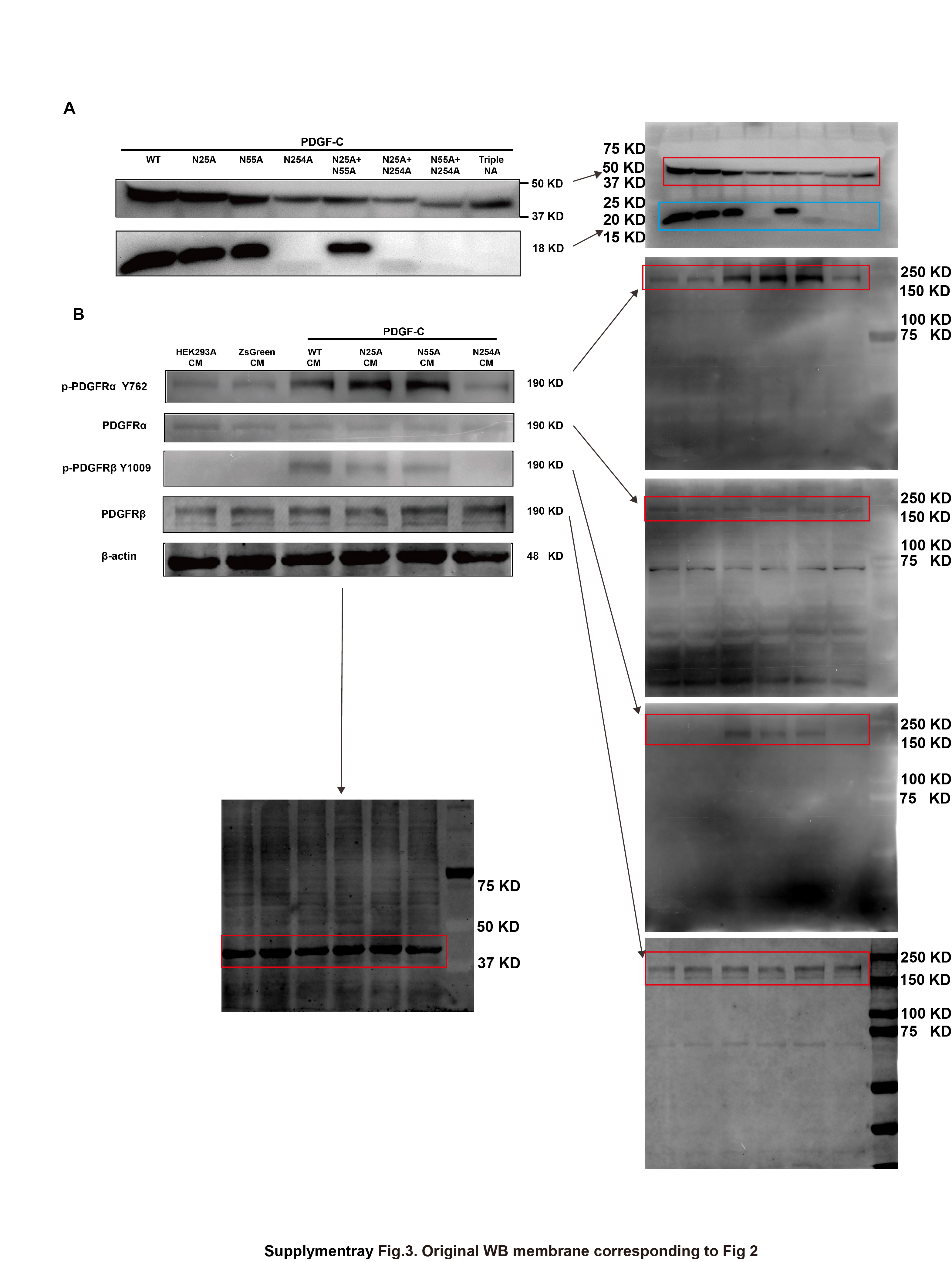

Supplement: Supplementary file 1 [file Image3.JPEG]

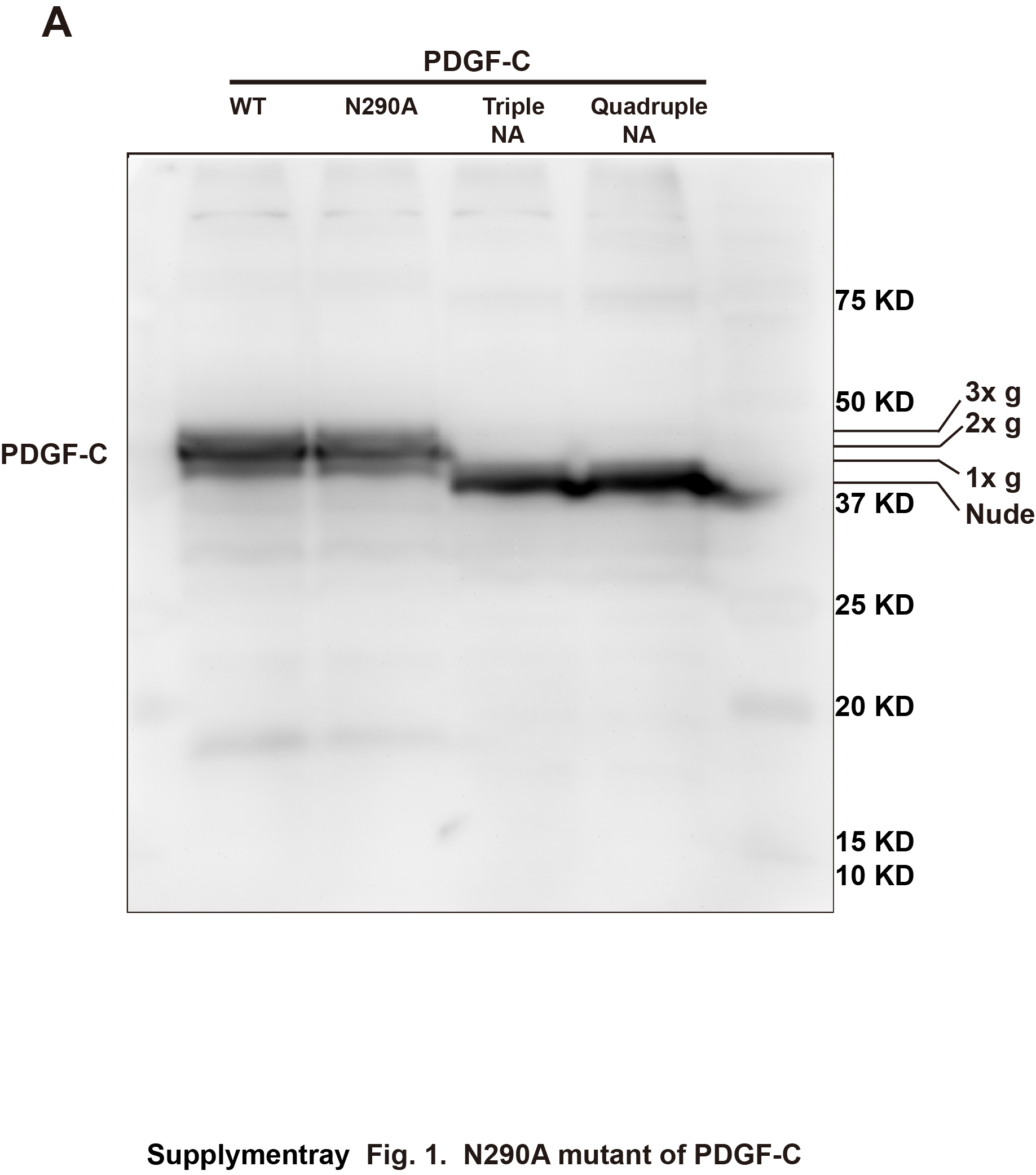

Supplement: Supplementary file 2 [file Image1.JPEG]

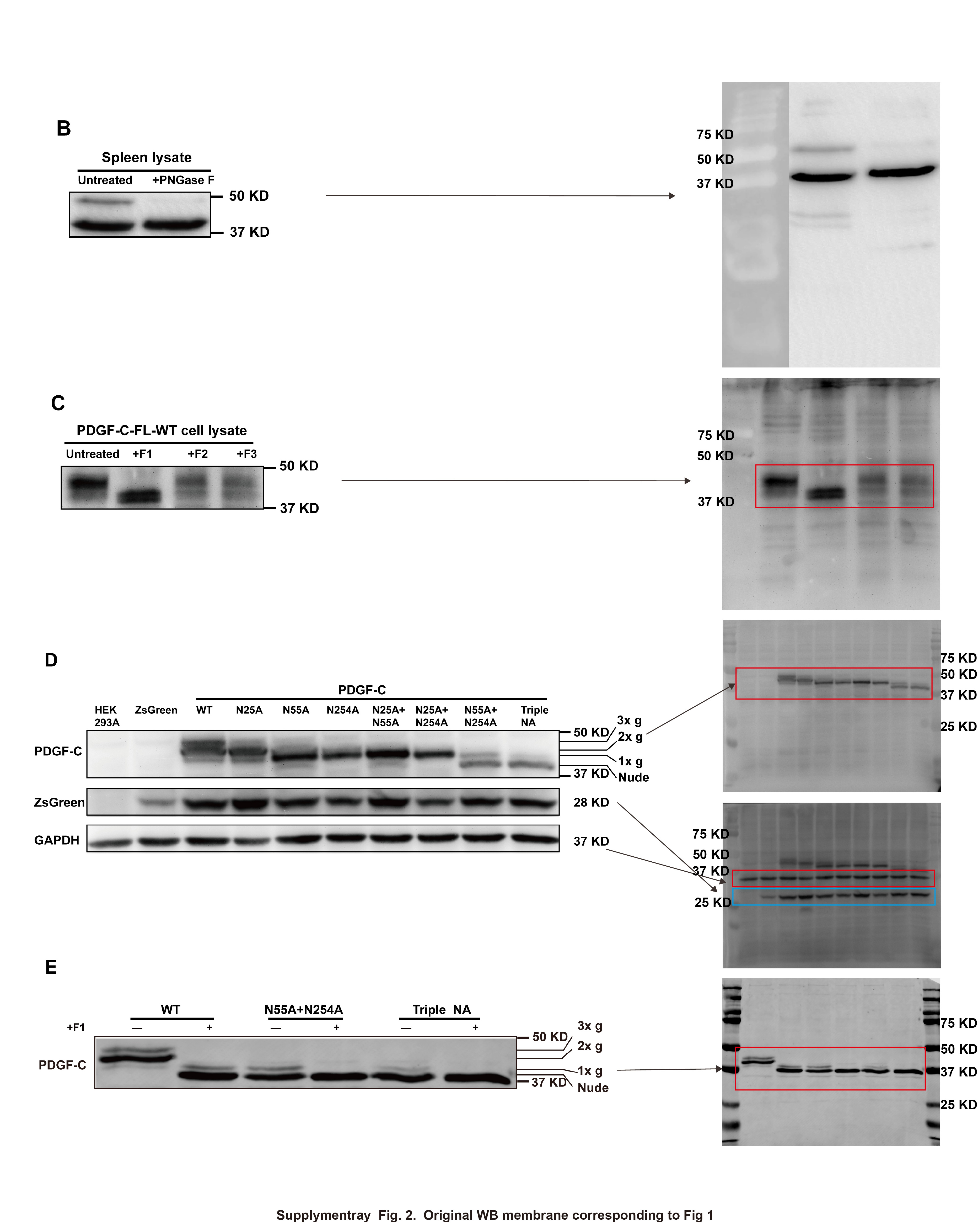

Supplement: Supplementary file 3 [file Image2.JPEG]
